# Gold Dissolution from Ore with Iodide-Oxidising Bacteria

**DOI:** 10.1038/s41598-019-41004-8

**Published:** 2019-03-12

**Authors:** San Yee Khaing, Yuichi Sugai, Kyuro Sasaki

**Affiliations:** 1 Department of Earth Resources Engineering, Graduate School of Engineering, Kyushu University, 8190395 Fukuoka, Japan; 2 Department of Earth Resources Engineering, Faculty of Engineering, Kyushu University, 8190395 Fukuoka, Japan

## Abstract

Gold leaching from ore using iodide-iodine mixtures is an alternative to gold cyanidation. This study evaluated the ability of iodide-oxidising bacteria to solubilise gold from ore that was mainly composed of gold, pyrite, galena, and chalcopyrite. Eight bacterial strains were successfully isolated from brine. Those strains were incubated in a liquid culture medium containing ore with a gold content of 0.26 wt.% and pulp density of 3.3 w/v% to evaluate their abilities to mediate the dissolution of gold. The gold was solubilised completely within 30 days of incubation in the iodine-iodide lixiviant solution generated by three bacterial strains. One strain, in particular, completed the dissolution of gold within 5 days of incubation and was identified as a member of the genus *Roseovarius*. Thus, the possibility of bacterial gold leaching using iodide-oxidising bacteria was successfully demonstrated. Bioleaching gold with iodide would likely be more environmentally sustainable than traditional cyanide leaching. Further research is required to evaluate the techno-economic feasibility of this approach.

## Introduction

Gold cyanidation remains a major gold-leaching technology in most gold mining operations around the world because of its highly effective gold recovery and economic efficiency. However, major challenges related to gold cyanidation include environmental problems caused by the extremely toxic nature of cyanide. Several gold-leaching agents alternative to cyanide have been discovered and suggested by several researchers^1–5^. Of several proposed alternatives, halides (chloride, bromide and iodide) are best known for high gold-leaching efficiency and low environmental impacts compared with other agents^6^. Among those halogens, iodine forms more stable complex ions with gold in aqueous solutions because gold iodate complex ions have lower redox potential compared with other halogenated gold complex ions [${{\rm{E}}}_{({{\rm{A}}{\rm{u}}}^{+}/{\rm{A}}{\rm{u}})}^{0}=1.83\,{\rm{V}}$, ${{{\rm{E}}}^{0}}_{([{{\rm{A}}{\rm{u}}{\rm{C}}{\rm{I}}}_{2}{]}^{-}/{\rm{A}}{\rm{u}})}=1.15\,{\rm{V}}$, ${{{\rm{E}}}^{0}}_{([{{\rm{A}}{\rm{u}}{\rm{B}}{\rm{r}}}_{2}{]}^{-}/{\rm{A}}{\rm{u}})}=0.96\,{\rm{V}}$, ${{{\rm{E}}}^{0}}_{([{{\rm{A}}{\rm{u}}{\rm{l}}}_{2}{]}^{-}/{\rm{A}}{\rm{u}})}=0.576\,{\rm{V}}$, ${{{\rm{E}}}^{0}}_{([{{\rm{A}}{\rm{u}}{\rm{l}}}_{4}{]}^{-}/{\rm{A}}{\rm{u}})}=0.57\,{\rm{V}}$ (vs. SHE at 25 °C)]^6,7^. The fundamental aspects of gold leaching using iodide-iodine solutions have been described and discussed in the literature^7–10^. In an iodide-iodine mixture, iodine (I_2_) reacts with iodide (I^−^) in aqueous solution to form triiodide (I_3_^−^) according to reaction (1):

1 $${{\rm{I}}}^{-}+{{\rm{I}}}_{2}\to {{{\rm{I}}}_{3}}^{-}$$

Gold can be oxidised in an iodide-iodine mixture to gold (I) diiodide and/or gold (III) tetraiodide according to reactions (2) and (3):

2 $$2{\rm{Au}}+{{{\rm{I}}}_{3}}^{-}+{{\rm{I}}}^{-}\to 2{[{{\rm{AuI}}}_{2}]}^{-}$$

3 $$2{\rm{Au}}+3{{{\rm{I}}}_{3}}^{-}\to 2{[{{\rm{AuI}}}_{4}]}^{-}+{{\rm{I}}}^{-}$$

The dissolution rate of gold is directly proportional to the concentrations of iodine and iodide, unaffected by a change in pH between 2 and 10^9,10^, in contrast to gold cyanidation, which is carried out within a specific range of alkaline pH between 10 and 11.

Microorganisms are used in the extraction of base and precious metals from primary ores and concentrates through bioleaching and biooxidation. Bioleaching refers to microbially catalysed solubilisation of metals from solid materials^11^. Bioleaching has been successfully applied in the commercial extraction of valuable metals such as copper^12–17^, uranium^18,19^ and zinc^20^ from low-grade ores. Biooxidation has been used as a pre-treatment to dissolve sulphide minerals from refractory gold ores before cyanide leaching^21–23^.

A number of bacteria have been shown to oxidise I^−^ and help to regenerate the iodide-iodine lixiviant by oxidising I^−^ to I_2_^24^. A heterotrophic, Gram-negative bacterium “*P. iodooxidans*” can oxidise I^−^ to I_2_^25^, through an extracellular peroxidase with hydrogen peroxide (H_2_O_2_) as an electron acceptor^26,27^:

4 $${{\rm{H}}}_{2}{{\rm{O}}}_{2}+2{{\rm{I}}}^{-}+2{{\rm{H}}}^{+}\to {{\rm{I}}}_{2}+2{{\rm{H}}}_{2}{\rm{O}}$$

Recent studies^24,28,29^ have indicated that certain bacterial strains, such as *Roseovarius tolerans*, *Rhodothalassium salexigens* and *Roseovarius* spp., oxidise I^−^ to I_2_, and that the iodide-oxidising reaction was mediated by an extracellular oxidase that requires oxygen^24^:

5 $$4{{\rm{I}}}^{-}+{{\rm{O}}}_{2}+4{{\rm{H}}}^{+}\to 2{{\rm{I}}}_{2}+2{{\rm{H}}}_{2}{\rm{O}}$$

Although the oxidation of I^−^ by oxygen as an electron acceptor is energetically favourable, the extracellular nature of the enzyme implies that energy conservation by this reaction is not possible^24^. Iodide-oxidising bacteria (IOB) seem to prefer iodide-rich environments^24^.

Natural gas brine samples used in this study were collected from a natural gas field in Chiba Prefecture, Japan. The brine samples contained not only natural gas but also iodide at high concentrations, such as 120 ppm, which is more than 1,500 times the iodide concentration of seawater. The presence of IOB in the iodide-rich brine water collected from natural gas fields has been reported^24,29^.

Thus, IOB can be isolated from brine water and are capable of oxidising I^−^ into I_2_, which in turn forms I_3_^−^ in the culture solution by the chemical reactions (1) to (3). Kaksonen *et al*.^30^ have proposed the use of biogenic iodide-iodine as a lixiviant solution for gold leaching and the regeneration of the lixiviant with iodide-oxidising microorganisms.

Even though bioleaching of gold with biogenic iodine-iodide lixiviant has been suggested, it is yet to be practically demonstrated. To the best of our knowledge, this is the first report examining the bioleaching of gold from gold ores using IOB. The principal objective of the present study is to examine the feasibility of bacterial leaching of gold ores using IOB. This study addresses the isolation of IOB from the natural environment, screening of effective IOB for gold dissolution, and demonstration of gold dissolution from gold ore using IOB-generated iodine-iodide lixiviant.

## Results

### Isolation of IOB

**Figure 1:**
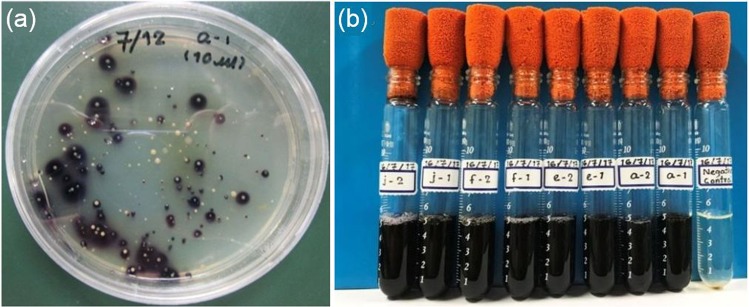
Agar plates for isolating IOB and liquid culture experiments for screening IOB that can grow under high-iodideconcentration conditions. (**a**) The inoculated solid culture medium which consisted of the marine broth, agar, potassium iodide and starch after incubation for a week at 30 °C. The colour of the culture medium around the colonies of IOB became purple because of the iodine-starch reaction; colonies of IOB can therefore be easily found. (**b**) The inoculated liquid culture solution including the marine broth, potassium iodide and starch after incubation of colonies of IOB isolated from the solid medium shown in (**a**) for 30 days at 30 °C. The colour of the culture solution became purple because of the iodine-starch reaction, whereas the colour of the negative control (without inoculation of IOB) did not change.

After incubation of brine samples for 7 days on a solid culture medium, colouration to purple was observed around some colonies, as shown in Fig. 1a. This colouration was caused by a chemical reaction between starch and triiodide. Triiodide formed through a chemical reaction between iodide and iodine, as shown by chemical reaction (1), and iodine formed through the oxidation of iodide by IOB. Among those colonies, eight bacterial strains were isolated from the solid culture media based on differences in shape, size, colour and texture. These colonies were named a-1, a-2, e-1, e-2, f-1, f-2, j-1 and j-2, respectively.

### First screening of IOB capable of growing and oxidising iodide in an environment with high-iodide concentration

The eight bacterial strains, isolated as described above, were incubated in a liquid culture medium with a high-iodide concentration (>20,000 ppm) to select competent IOB that could grow and oxidise iodide in such a high-iodide concentration environment. Figure 1b shows photographs of the culture solutions that had been incubated at 30 °C for 30 days.

All of these solutions except for the non-inoculated control had changed colour to purple because of the reaction between starch and triiodide. The initial bacterial cell population was 3 × 10^6^ cells/mL, and the bacterial cell counting after incubation indicated that populations of all eight strains had increased to more than 1 × 10^8^ cells/mL. This result shows that all eight bacterial strains could grow and oxidise iodide in a high-iodideconcentration environment. Therefore, all of these strains were used in the next screening step.

### Second screening of competent IOB capable of solubilising gold from gold ore

Next, the eight bacterial strains were incubated in the liquid culture medium containing the marine broth, potassium iodide and sulphide gold ore sample to evaluate their abilities to extract gold from a real gold ore sample. X-ray diffraction (XRD) analysis indicated that the gold ore was mainly composed of quartz, galena, pyrite, chalcopyrite and gold. The chemical compositions of oxides and elements in the original gold ore and solid residue collected from culture solution after the incubation were determined by X-ray fluorescence (XRF) analysis. The results of the XRF analysis are shown in Table 1. The original gold content of the ore was approximately 0.26 wt.%. The decrease in the gold content in the solid residue collected from the culture solution after the incubation was observed in all the culture solutions in which the eight strains were incubated for 30 days at 30 °C, whereas decrease in the gold content in the negative control was not observed. In particular, the gold contents in the solid residue collected from the culture solutions of three strains (a-1, e-2 and f-1) were completely reduced.

**Table 1:** Chemical compositions (wt.%) of oxides and elements obtained by XRF analysis of the original gold ore (before incubation) and the solid residue (after incubation).

Sample type	Bacterial strain	Chemical compositions of oxides and elements, wt.%												
		SiO_2_	SO_3_	Pb	Al_2_O_3_	Fe_2_O_3_	CaO	K_2_O	ZnO	Au	MgO	Ag	Cr_2_O_3_	LOI
Original ore sample before the microbial treatment	—	78.6	4.57	3.37	3.47	3.12	2.92	1.64	0.82	0.26	0.24	0.20	0.16	0.61
Ore samples (solid residue) after the microbial treatment with each bacterial strain	a-1	85.6	4.07	2.3	2.15	1.97	1.49	1.07	0.49	0.00	0.16	0.14	0.24	0.32
	a-2	82.6	2.82	3.43	2.63	2.58	2.75	1.38	0.80	0.12	0.25	0.14	0.20	0.32
	e-1	83.1	4.63	3.00	2.18	2.35	2.09	1.17	0.66	0.10	0.15	0.13	0.15	0.29
	e-2	82.8	5.07	2.58	2.35	2.36	2.28	1.30	0.63	0.00	0.16	0.13	0.11	0.23
	f-1	83.8	5.03	3.00	2.09	1.82	1.48	1.23	0.56	0.00	0.13	0.17	0.33	0.36
	f-2	79.1	6.23	3.30	2.50	2.90	2.31	1.56	0.90	0.18	0.23	0.00	0.27	0.40
	j-1	85.3	5.05	2.4	1.97	1.46	1.62	1.09	0.38	0.03	0.13	0.09	0.24	0.24
	j-2	83.7	4.40	2.73	2.07	2.54	1.70	1.09	0.62	0.04	0.16	0.13	0.21	0.61
	NC*	83.1	4.82	2.78	2.29	1.98	2.27	1.13	0.49	0.26	0.15	0.12	0.21	0.38

The Au content of the original ore sample used in this study was 0.26 wt.%. ^*^NC: Negative Control.

**Figure 2:**
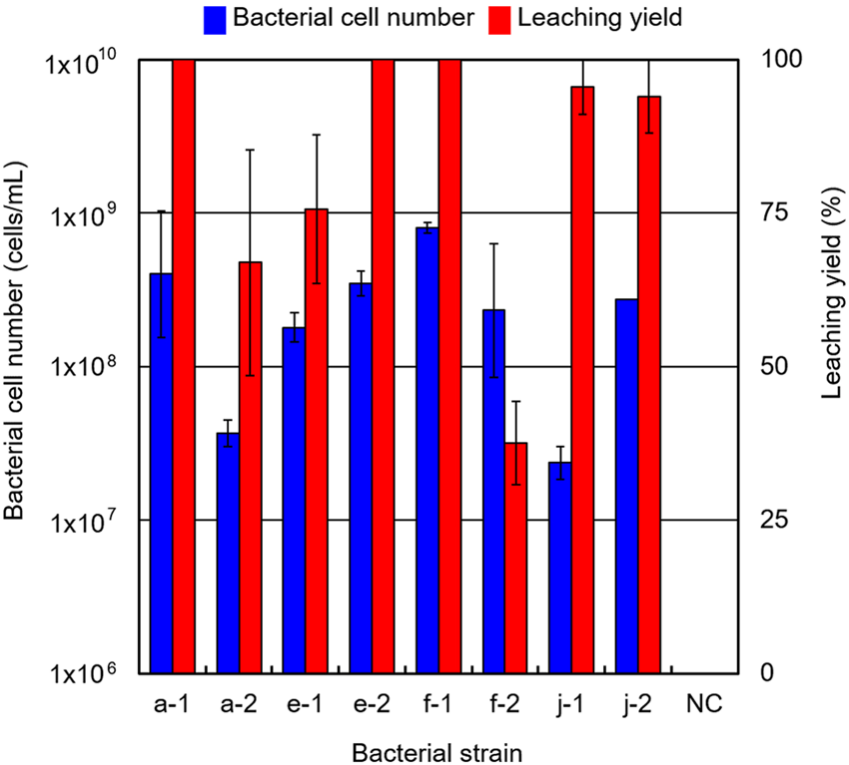
Bacterial cell numbers and leaching yield in the ore sample after 30 days. Bacterial cell number (blue bar) is indicated in cells/mL by the left vertical axis with a logarithmic scale and error bars. The leaching yield in the ore sample (red bar) is indicated in percentage by the right vertical axis with a linear scale and error bars. NC stands for the negative control without inoculation of bacteria. The pulp density used in this study was 3.3 w/v%. The leaching yield was calculated by equation (6) using the results of XRF analysis of the solid residue collected from the culture solution after 30 days of incubation.

Figure 2 highlights the bacterial cell numbers and the leaching yield (%), which were calculated based on the results of the XRF analysis and can be expressed as follows:

6 $${\rm{Leaching}}\,{\rm{yield}}=\frac{{\rm{Original}}\,{\rm{mass}}\,{\rm{of}}\,{\rm{gold}}\,{\rm{in}}\,{\rm{the}}\,{\rm{ore}}\,{\rm{sample}}-{\rm{Final}}\,{\rm{mass}}\,{\rm{of}}\,{\rm{gold}}\,{\rm{in}}\,{\rm{the}}\,{\rm{ore}}\,{\rm{sample}}}{{\rm{Original}}\,{\rm{mass}}\,{\rm{of}}\,{\rm{gold}}\,{\rm{in}}\,{\rm{the}}\,{\rm{ore}}\,{\rm{sample}}}\times 100\,( \% )$$

The bacterial cell numbers of all eight strains increased by over an order of magnitude in the liquid culture medium during incubation (Fig. 2). In particular, the bacterial cell number of strain a-1 increased to 5 × 10^8^ cells/mL, which was the largest increment in this study. A decrease in the residual gold content was observed for all bacterial strains except strain f-2. In particular, gold in the ore sample was decreased to zero by strains a-1, e-2 and f-1. As the decrease in residual gold content was not observed for the negative control, it can be inferred that the activities of the IOB caused a decrease in residual gold content.

ICP-MS analysis of dissolved gold in the culture solution was carried out to further confirm the leaching yield of gold from the solution. The ICP-MS analysis indicated that the gold concentrations in the culture solutions of strains a-1, e-2 and f-1 were 83 ppm, 86 ppm and 90 ppm, respectively. The leaching yield of gold was calculated from the gold mass in both the culture solutions and original ore sample. The leaching yields of the culture solutions of strains a-1, e-2 and f-1 were 92.1%, 95.5% and 99.9%, respectively. The leaching yields calculated from the results of the XRF analysis of the solid residues were 100%, as shown in Fig. 2; therefore, high consistency could be obtained between the analyses of both the solution and the solid residue. These results therefore suggest that the leaching yield calculated based on the results of the XRF analysis of the solid residues in this study are relatively accurate.

**Figure 3:**
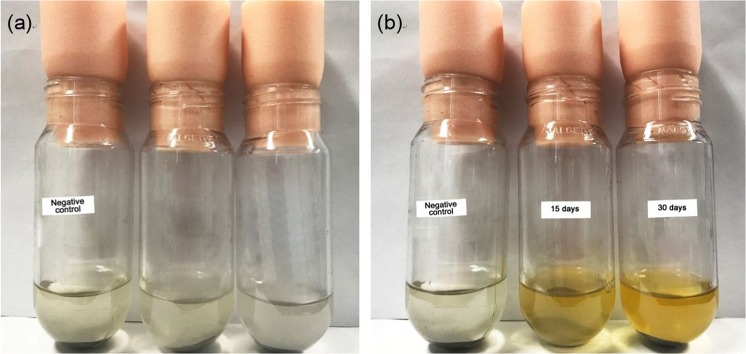
Liquid culture experiments for the second screening of IOB that extracted gold from the ore sample in the culture solution. (**a**) Marine broth liquid medium with potassium iodide just after inoculation of the liquid culture medium of IOB from the pre-culture solution with 0.5 g of sterile ground ore samples at 30 °C. (**b**) The same culture solution after 15 days and 30 days of incubation. The colour of the culture solution became yellow and deep yellow after 15 days and 30 days, respectively; however, the colour of the negative control (without inoculation of IOB) did not change. During incubation, the IOB were capable of oxidising iodide (I^−^) into iodine (I_2_), which in turn formed triiodide (I_3_^−^) in the culture solution by chemical reaction (1). Subsequently, triiodide in the culture solution extracted gold from the ore sample according to chemical reactions (2) and (3). Pulp density used in this experiment was 3.3 w/v%.

Figure 3 shows photographic images of the negative control and culture solution of strain a-1 taken after the passage of 0 days, 15 days and 30 days after the start of the experiments. The colour of the culture solution changed from clear and colourless to deep yellow as the incubation period proceeded, whereas the colour of the negative control was not changed. This colouration suggests that I_2_ was generated by IOB in the culture solution because the colour of triiodide generated by chemical reaction (1) is yellow^24^. Consequently, gold was dissolved into the culture solution from the ore sample. Also, the bacterial cell number of strain a-1 (Fig. 3b) increased to 5 × 10^8^ cells/mL after 30 days. On the basis of these results, the three bacterial strains that successfully decreased the gold content of the ore sample to zero, strains a-1, e-2 and f-1, were selected as potential bacterial strains for leaching gold from the ore sample and were subjected to the next screening step.

Finally, Table 2 summarises the enrichment cultures and screening results of leaching experiments for the eight bacterial strains by showing the morphology, leaching yield and similarity to identified bacteria. The eight bacterial strains were identified by analysing the sequences of their 16S rRNA genes. The results of the analysis indicate that all eight strains belong to the genus *Roseovarius*, with sequence similarity of 92% to 94%.

**Table 2:** Summary of characteristics of eight bacterial strains isolated as IOB through the enrichment cultures and subjected to the first and second screening experiments.

Strain	Colony morphology	Bacterial species	Similarity to identified bacteria	Leaching yield
a-1	Round and small	*Roseovarius* spp.	94%	100%
a-2	Round and small	*Roseovarius* spp.	92%	66.9%
e-1	Round and small	*Roseovarius* spp.	94%	75.7%
e-2	Round and small	*Roseovarius* spp.	94%	100%
f-1	Round and small	*Roseovarius* spp.	94%	100%
f-2	Round and small	*Roseovarius* spp.	94%	37.6%
j-1	Round and small	*Roseovarius* spp.	92%	95.6%
j-2	Round and small	*Roseovarius* spp.	92%	94.0%

### Third screening of competent IOB effective for fast gold leaching

**Figure 4:**
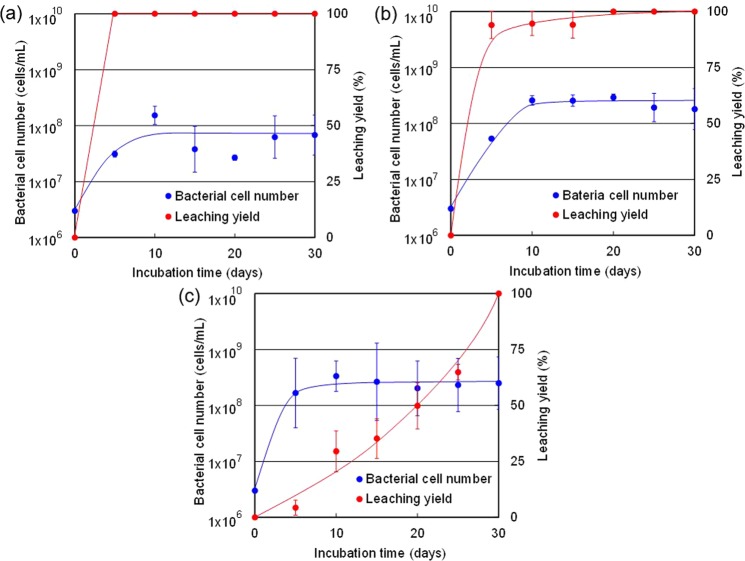
Dynamic changes of bacterial cell number and leaching yield of gold from the ore sample during the incubation experiments. Temporal changes of bacterial cell number (blue plots) and Au leaching yield in the ore sample (red plots) in the culture solution of bacterial strains (**a**) a-1, (**b**) e-2 and (**c**) f-1. The bacterial cell number is indicated by the left vertical axis in cells/mL with a logarithmic scale. The leaching yield is indicated by the right vertical axis in percentage with a linear scale.

Strains a-1, e-2 and f-1 were incubated in the same liquid culture medium under the same conditions to evaluate their effects on the leaching yield of gold from the ore sample. Figure 4 shows the temporal changes of bacterial cell number and leaching yield during the incubation experiments. Strain a-1 grew just after the start of the incubation experiment, and its bacterial cell number reached a maximum after 10 days of incubation. Thereafter, the bacterial cell number plateaued until 30 days. Initially, the bacterial cell number of strain a-1 was 3 × 10^6^ cells/mL but increased to 5 × 10^7^ cells/mL after 5 days and remained unchanged after 10–30 days of incubation. Likewise, the initial bacterial cell number of strain e-2 started from 3 × 10^6^ cells/mL but increased to 7 × 10^7^ cells/mL after 5 days, until it stabilised at a maximum cell number of 4 × 10^8^ cells/mL after 10–30 days of incubation. In the case of strain f-1, the bacterial cell number reached more than 2 × 10^8^ cells/mL after 5 days of incubation and changed insignificantly until 30 days.

Because the incubation experiment was carried out in closed system (batch system), the growth rate of bacteria was calculated using the experimental results obtained before the cell number reached a plateau. Accordingly, the growth rates of strains a-1 and e-2 were calculated using the experimental results obtained before 10 days, and that of strain f-1 was calculated using the experimental results obtained before 5 days. The growth rates of a-1, e-2 and f-1 were 0.393 day^−1^, 0.445 day^−1^ and 0.804 day^−1^, respectively. The growth of strain f-1 was the fastest in the three strains.

However, the leaching yield increased faster in the culture solutions of strains a-1 and e-2 compared with the culture solution of strain f-1. The leaching yield in the culture solution of strain a-1 increased to 100% after 5 days, whereas those in the culture solutions of e-2 and f-1 increased to 100% after 20 days and 30 days, respectively. After 30 days of incubation, no traces of gold were detected in the solid residues of all three samples collected from the culture solutions of a-1, e-2 and f-1, based on the XRF analysis.

Thus, three bacterial strains that generated iodine-iodide lixiviant solution that completely dissolved gold from the ore sample were successfully isolated through this study. In particular, one of these strains, named a-1, was selected as the most promising bacterial strain for gold leaching because it showed the highest capability for leaching gold from gold ore quickly, as shown in Fig. 4. These results demonstrate the potential for bacterial leaching of gold ore using IOB.

Iodine is not the only effective or important lixiviant for gold leaching; triiodide is as well. The behaviour of triiodide generation was therefore investigated in this study. The two bacterial strains a-1 and f-1, which were the most effective strain (a-1) and a slightly inferior strain (f-1) respectively, were incubated in a culture medium that did not contain the ore sample to observe the behaviour of triiodide generation. The ore sample was not put in the culture medium in this experiment to prevent the consumption of triiodide due to the chemical reaction between triiodide and gold in the ore sample and to understand the behaviour of triiodide generation.

**Figure 5:**
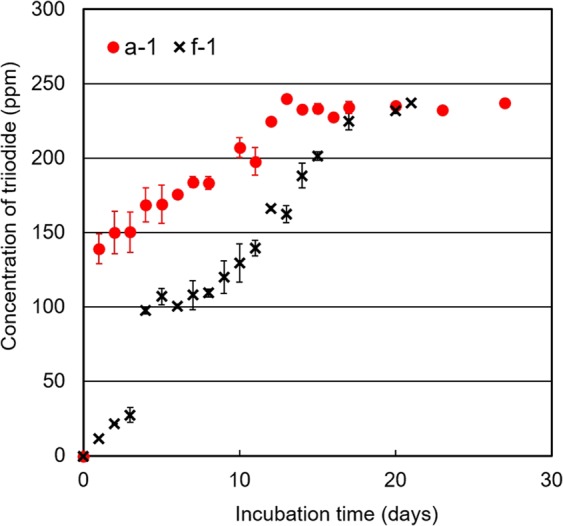
Variation of triiodide concentrations with incubation time. The generation of triiodide was evaluated based on measurement of the absorbance at 351 nm^48^ by UV-visible spectrophotometry, and the absorbance was converted to triiodide concentration by a calibration curve prepared using the triiodide standard solution. Strains a-1 and f-1 were the two effective bacterial strains that completely leached gold from the ore sample in the leaching experiment and were used in the determination of leaching yield with respect to time.

The concentration of triiodide in the culture solutions of both strains increased to 220–240 ppm, as shown in Fig. 5. The concentration of triiodide in the culture solution was high enough for gold to be completely leached from the ore sample used in this study. In particular, the concentration of triiodide in the culture solution of strain a-1 reached a plateau faster than that of strain f-1. Because the actual rate of triiodide generation is useful data for comparing the performance of selected strains, we compared the rate for each strain. Because the incubation experiment was performed in a closed system, the actual rate was calculated using the results obtained before the concentration of triiodide reached a plateau. Accordingly, the actual rates of triiodide generation by strains a-1 and f-1 were calculated using the experimental results obtained before 13 days and 17 days had elapsed after the start of the experiment, respectively. The actual rates of triiodide generation by strains a-1 and f-1 were 18.5 ppm day^−1^ and 13.2 ppm day^−1^, respectively. The behaviour of triiodide generation was consistent with the behaviour of the leaching yield; that is, the higher actual rate of triiodide generation, the greater leaching yield (%) of gold. To this effect, a primary step to experimentally demonstrate the possibility of gold leaching using IOB is to qualitatively assess the behaviour of triiodide generation.

**Figure 6:**
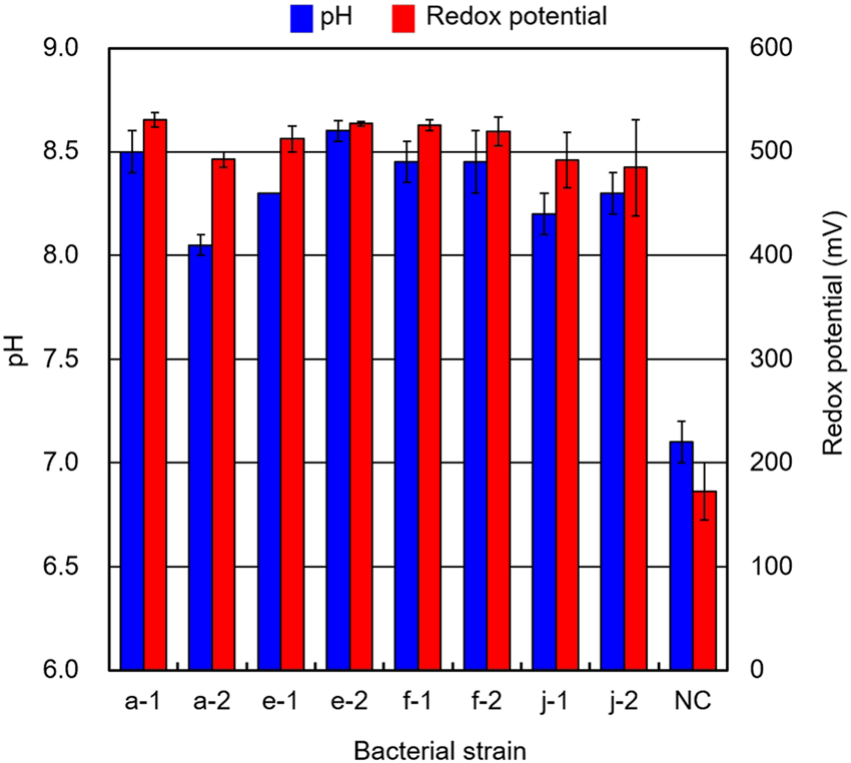
pH value and redox potential of the culture solution after 30 days. The pH value (blue bar) is indicated by the left vertical axis with an error bar. The redox potential (red bar) is indicated in mV by the right vertical axis with an error bar. NC stands for the negative control without inoculation of bacteria.

To understand the reactions and process variables of the IOB gold-leaching mechanism, measurements of pH and redox potential were carried out for the liquid medium. The initial pH of the medium was approximately 8.2. The pH of the medium varied from 8.0 to 8.8 during the experiments. Also, the redox potential of the culture solution ranged from 498 mV to 547 mV, as shown in Fig. 6. The values of pH and redox potential continued to increase during the incubation experiments. The increasing trends differed depending on the growth of IOB and the iodine-iodide reaction in the culture solution. Both the pH and redox potential of the culture solution of strain a-1 increased continuously before 5 days from the start of the incubation. The pH increased to 8.8 in that period from 8.0, the original pH of the culture medium. The redox potential increased to 547 mV in that period from 522 mV, the original redox potential of the culture medium. Thereafter, both pH and redox potential remained approximately constant.

## Discussion

In this study, eight bacterial strains were successfully isolated from brine. Those strains were incubated in a liquid medium containing ground ore with 0.26 wt.% Au and 3.3 w/v% pulp density, and their abilities to leach gold from ore were evaluated. Decrease in residual gold content from the ore was observed for all bacterial strains except strain f-2.

All eight bacterial strains isolated as IOB in this study were identified as the same genus, *Roseovarius*, by analysing 16S rRNA gene sequences, as shown in Table 2. This genus has been previously reported as a kind of IOB capable of oxidising iodide to iodine^24^. Although all eight bacterial strains belonged to the same genus, the results of the incubation experiments differed between the strains. These eight bacterial strains may belong to different species, which could be identified through full sequencing analysis; therefore, differences in their abilities to dissolve gold from the ore sample were associated with differences between the species or strains. In particular, three strains (a-1, e-2 and f-1) showed high capacity to generate iodine-iodide lixiviant solution that completely solubilised gold from the ore sample within 30 days of incubation.

Strain a-1 also grew until 5 days of incubation, and the bacterial cell number remained approximately constant after that. On the basis of comparison of the similarity in the increasing trends of pH, redox potential and bacterial growth, the increase in bacterial activity caused the concomitant rise in pH and redox potential. Similar results were also obtained in the experiments incubating strains e-2 and f-1.

Because gold dissolution is insensitive to pH over the range of pH 2–10^31^ and the dissolution rate depends upon the concentrations of iodide and iodine^9,32,33^, microbial activities therefore played a critical role in accelerating dissolution of gold from ore in the bioleaching using IOB. On the basis of the pH and redox potential conditions, with near-neutral pH and low redox potential values, the type of gold in the solution could be designated as AuI_2_^−^ ^32,33^.

Gold concentration in the culture solution of strain a-1 was quantified after 30 days of incubation to evaluate the mass balance of gold in the culture system. On the basis of the results of ICP-MS and XRF analyses, increase in the concentrations of gold in the solution relative to decrease in the gold contents of ore samples was observed to be balanced. The growth of strain a-1 stabilised after 5–10 days of cultivation (Fig. 4). Iodine can therefore be assumed to be generated by IOB from the logarithmic growth phase to the early stage of the stationary phase. The concentration of triiodide was still increasing after the 5th day, whereas the gold was completely leached into the culture solution by the 5th day. The additive amount of potassium iodide in this experiment can therefore be assumed to be excessive. Thus, the optimum amount of iodide should be considered depending on the gold grade of the ore.

The leaching rate was higher for strains a-1, e-2 and f-1, whereas the growth rate was higher for strains f-1, e-2 and a-1. Strain a-1 was assumed to have higher iodide oxidation capability than the other two strains. Iodine was therefore generated at a higher rate in the culture solution of strain a-1, and the leaching rate became higher. However, the growth of a-1 might have been inhibited by iodine generated by that strain itself at an early stage of the incubation experiment. Zhao *et al*. investigated the negative influence of iodine on the growth of three IOB strains, which were all belonging to *Roseovarius* spp., through the incubation experiments using marine broth medium containing molecular iodine^34^. They showed that the growth of their IOB strains which were exposed to 1–2 ppm, 5 ppm and 10 ppm of iodine was suppressed to 70%, 50% and 20% respectively as compared with the cases where they were incubated without molecular iodine.

They determined the iodine concentration of the culture solution according to the following equilibrium equation:

7 $$[{{\rm{I}}}_{2}]=\frac{[{{\rm{I}}}_{3}^{-}]}{{K}_{{\rm{C}}}[{{\rm{I}}}^{-}]}$$

where [I^−^], [I_2_], [I_3_^−^] and *K*_C_ are the equilibrium concentration of iodide, molecular iodine, triiodide and equilibrium constant respectively. The iodine concentration in the culture solution of a-1 of this study can be calculated using the same equilibrium equation with the *K*_C_ in pure water which was found to be 626 L/mol at 30 °C, which is in close agreement with the value reported by Palmer *et al*.^35^. The iodine concentration was approximately calculated as 2.3 ppm by using the concentration of iodide and triiodide at 15 days after the start of the incubation of the strain a-1. According to the report of Zhao *et al*. described above, it can be assumed that the growth of the strain a-1 was suppressed to 70% under the condition of such iodine concentration in this study. Because of this negative impact of iodine on the strain a-1, the growth rate of a-1 was the lowest whereas the leaching rate of a-1 was the highest. In contrast, f-1 had lower capability of iodide oxidation and it generated iodine at a lower rate. Thus, the growth of f-1 was less inhibited. The growth rate of f-1 was therefore higher, but the leaching rate was lower.

According to the results of the XRF analysis of the ore samples before and after incubation, the contents of other trace elements such as Al, Pb and Ag in the ore samples were also decreased after incubation. This finding indicates that trace elements were also dissolved into the culture solution from the ore sample. Those elements often affect the growth and metabolism of bacteria. Although the adverse effects of those elements on the activities of IOB were not investigated in this study, it is considered that the activities of f-2 during the second screening might have been affected by those trace elements^36–38^.

An experiment on cyanide leaching of finely disseminated gold ore (104 µm grain size, pH ≥ 10.5) indicated that higher gold dissolution (97.5%) occurred after about 40 hours of leaching time^39^. Another bioleaching study indicated that the cyanogenic bacteria *Pseudomonas plecoglossicida* solubilised gold from shredded printed circuit boards with 69% dissolution in 80 hours^40^. A two-step bioleaching process to leach gold from electronic waste indicated that *Chromobacterium violaceum* was capable of leaching 69% of gold and that a mixture of *Chromobacterium violaceum* and *Pseudomonas aeruginosa* exhibited 73% gold leaching within 7 days^41^. The present study demonstrated that gold can be solubilised at a greater yield (i.e. 95–100% leaching yield) from high-grade ground ore (average particle size of 75 µm) within 5–30 days of the bioleaching experiment using the IOB-generated iodine-iodide lixiviant. The iodide-iodine system does not normally oxidise metal sulphides; therefore, excessive reagent consumption can be avoided in the leaching process and the process is suitable for sulphide ores^42^. Metal sulphides are oxidised during the cyanidation process, and cyanide is consumed by reaction with metal sulphides. Thus, cyanide is used more during the leaching process. In the case of the iodide-iodine system, iodine and iodide are not consumed by oxidation of metal sulphides. Therefore, the iodine-iodide system has an advantage over the cyanidation process.

Leaching of gold by the iodine-iodide system can be carried out in a wide pH range between 2 and 10^31^, in contrast to cyanide leaching, which is normally carried out under relatively restricted alkaline conditions (pH 10 to 11). Iodine can act as an oxidant, and no other oxidant is required in this bioleaching experiment^32^. IOB was found to be much more abundant in the iodide-rich brine waters than in natural seawater, which indicates that they can be active in slightly alkaline conditions. IOB growth could possibly occur in a pH range of 4.5 to 8.5^43^. In addition, iodine can leach gold from its ore at low concentrations, penetrating rocks particularly well, and does not adsorb on gangue particles to any great extent, allowing for excellent recovery of the reagent and reducing the cost of the process^44^.

Bioleaching of gold ore using IOB may be considered an environmentally friendly process because of its lower toxicity than conventional cyanide. However, the leaching (contact) time may not be as fast as that of direct chemical leaching by cyanide, which requires contact times of 24–72 hours for gold ore^32^. In practice, the costs of the reagent (potassium iodide) and nutrients (marine broth) may be expensive, which may be a cost factor to consider for bioleaching operation using IOB.

The possibility of bacterial gold leaching using IOB was successfully demonstrated in the present study. A direct comparison cannot be made between the results of the present study and those of other chemical- and bioleaching studies. Reasons for this include differences in the nature of the treatments, growth media, microorganisms, and compositions of ores. However, bacterial leaching in this study is shown to be effective and offers better conditions with considerable advantages over other biocyanidation and conventional cyanidation processes.

This study could be improved by doing more intensive experiments using a column and/or bioreactor to examine the relationship between microbial community function and leaching behaviour, controlling the microbial generation of iodine and the leaching mechanisms, optimising the iodine-iodide molar ratio and their concentrations, using oxidants to minimise loss of iodine during leaching operation to find the best practical conditions for effective gold dissolution, and regenerating the lixiviants for economic efficiency. Although this work was carried out to leach gold successfully from high-grade gold ore, it is hoped that with further research this technique could also be adopted to treat low-grade gold ore. Thus, studies on these parameters should be continued in future works to improve the technique and optimise conditions for effective gold leaching.

## Materials and Methods

### Enrichment and Isolation

#### Sampling of the ore and brine

A gold ore rock sample was collected from the Modi Taung gold mine in central Myanmar. Natural gas brine waters (two samples) were collected from Chiba Prefecture, Japan. These samples were kept at room temperature in sterile plastic bottles until use. Iodide concentrations in the brine water samples were measured at more than 120 ppm by ion chromatography^45^.

#### Enrichment and isolation of IOB

A solid culture medium was used for isolation of IOB from the brine. Then, incubation was performed using a liquid solution composed of Difco™ marine broth 2216 (37.4 g/L), agar powder (20 g/L), soluble starch (1.2 g/L) and potassium iodide (1.0 g/L) was autoclaved at 121 °C for 20 minutes. Next, 15 mL of the sterilised solution was poured into a sterilised petri dish. After the culture medium was solidified by cooling, 100 µL of the brine was spread onto the surface of the solid culture medium using a sterilised spreader. The culture medium was incubated at 30 °C under aerobic conditions and observed daily.

Colonies of bacteria could be found on the solid culture medium after 5–10 days of incubation. The colonies of IOB could be clearly distinguished because the colour of the culture medium at the periphery of the colonies changed to purple. Single colonies of IOB were picked and streaked out onto another identical solid culture medium to obtain pure isolates of IOB. This work was repeated twice to obtain pure isolates of IOB.

### Screening tests

#### Incubation experiment using liquid culture media

Two kinds of liquid culture media were used for the first and second screenings of effective isolates of IOB. The liquid culture medium used for the first screening was composed of Difco™ marine broth 2216 (37.4 g/L), soluble starch (1.2 g/L), and potassium iodide (21.8 g/L). Addition of high KI concentration into the medium was performed to successfully isolate and enrich IOB because they prefer high-iodideconcentration environment^24^. This liquid culture medium was autoclaved at 121 °C for 20 minutes. After the medium was cooled, single colonies of pure isolates obtained from previous isolation were inoculated into the liquid culture medium and incubated at 30 °C under aerobic conditions. A non-inoculated control was also prepared. After 4 weeks of incubation, the capabilities of the colonies for growth under the condition of high-iodide concentration (>20,000 ppm) and for oxidising iodide were evaluated. IOB growth was evaluated by counting bacterial cell numbers in the culture solution. Iodide oxidation capability was evaluated by observing the changing colour of culture solution into purple, which was caused by the iodine-starch reaction^46^. The isolates that were evaluated to be capable of growth and iodide oxidation were subjected to the second screening.

The liquid culture medium used for the second screening contained Difco™ marine broth 2216 and potassium iodide with the same concentration as that in the liquid culture medium used for the first screening. Fifteen millilitres of the solution was poured into a glass tube and autoclaved at 121 °C for 20 minutes. The IOB isolates were pre-cultured in a liquid culture medium containing only marine broth with the same concentration as described above, at 30 °C under aerobic conditions. This previously cultured solution of the isolate was inoculated into the culture medium to be used for secondary screening. In addition, the gold ore powder sample was wrapped in aluminium foil and sterilised in a furnace at 140 °C for 4 hours. After cooling, 0.5 g of the sterilised ore was put into 15 mL of the culture medium.

In the third screening, twelve bottles with the same culture medium were prepared for this experiment. Six of the bottles were incubated, and the other six were used as non-inoculated controls. The bottles were incubated at 30 °C for 30 days. One inoculated bottle and one uninoculated bottle were sequentially sampled every 5 days. The ore and solution were separated by filtration using a membrane filter with a pore size of 0.2 µm, and the ore was washed with pure water, dried at 50 °C and subjected to XRF analysis (ZSX Primus II, Rigaku Corporation, Tokyo, Japan) to evaluate the gold content.

The pH and redox potential of the filtrate were measured at room temperature using a handheld ion/pH meter (IM-32P, DKK-TOA Corporation, Tokyo, Japan) with an ion selective electrode. The reference electrode was a silver-silver chloride electrode in 3.3 mol/L solution of potassium chloride. The gold concentration in the solution was quantified by ICP-MS after the solution was pre-treated as follows. The solution was thermally decomposed with sulphuric acid and nitric acid^47^. The decomposition product was then dissolved completely in the aqua regia solution. After the post-aqua regia solution was filtered with a filter pore size of 0.45 μm, it was subjected to ICP-MS analysis. Extra pure water was added to the solution to maintain a constant volume. Subsequently, the concentration of gold in the solution was quantified through ICP-MS analysis.

Lastly, the methodology of the incubation experiment for the third screening was the same as that for the second screening. However, in the case of the third screening, the data were obtained every 5 days. Solid samples were analysed for each time point (i.e. 5 days, 10 days, 15 days, 20 days, 25 days and 30 days) using replications for the leaching experiment.

### Analytical methods

#### Cell counting

Cells were counted using a Petroff-Hausser counting chamber and a phase-contrast microscope, the EVOS XL Core Cell Imaging System provided by Thermo Fisher Scientific Inc. (Waltham, Massachusetts MA, USA). Replicate counts were made in accordance with the instruction of the counting chamber. Then, the isolates of IOB were incubated at 30 °C under aerobic conditions for 30 days.

#### XRF and ICP-MS analysis

This gold ore rock was crushed and ground to an average size of 75 µm or less using the CMT Vibrating Sample Mill (TI-100). The ground ore sample was used for the incubation experiments. The mineral composition and chemical composition were determined using XRD and XRF analysis, respectively. The mineral composition was analysed using a Rigaku RINT-2100 Diffractometer. The analysis was done using Cu Kα radiation at 40 kV and 20 mA, and count data for the random powder mounts were collected from 2°–65° two-theta at a scanning rate of 2° per minute. The chemical composition of the gold ore was measured from pressed pellets using a ZSX Primus II X-ray fluorescence spectrometer. The experiment was performed with an accelerating potential of 15 kV, a beam current of about 10 mA, and a beam diameter of 3 µm.

The soluble Au in the solution was measured by ICP-MS analysis. However, because of financial limitations, we could measure the Au content in only three solution samples in which the residual gold in the ore was not detected after 30 days of incubation. Because good balance between the gold concentration in the solution and gold content in the original ore sample could be obtained, the leaching yield could be evaluated by measuring the residual gold in the ore sample.

#### Identification of microbial species

Microbial species isolated from the formation water were identified through 16S rRNA gene sequence analysis. Each isolated strain of IOB was incubated on the solid culture medium as described above. Bacterial DNA was extracted from one colony of each IOB on the solid medium using an UltraClean Microbial DNA Isolation Kit (MO BIO Laboratories, Inc., USA) according to the manufacturer’s instructions. Extracted DNA was amplified by PCR targeting of the 16S rRNA genes using Premix Taq™ Hot Start Version (TAKARA BIO Inc., Japan) and primers. Almost all regions of the eubacterial 16S rRNA genes were amplified using universal primers (forward EU27f [5′-AGAGTTTGA TCCTGGCTCAG-3′], reverse EU1525r [5′-AAAGGAGG TGATCCAGCC-3′]). The thermal cycle profile for the PCR was as follows: initial denaturation at 94 °C for 2 min; 30 denaturation cycles at 94 °C for 1 min, annealing at 54 °C for 2 min, extension at 72 °C for 2 min; final extension at 72 °C for 7 min, and cooling at 4 °C. The DNA in the PCR product was purified using QIA quick PCR Purification Kit (QIAGEN, the Netherlands). The purified DNA was sequenced using a MicroSeq 500 16S rRNA gene Sequencing Kit (Applied Biosystems Inc., USA). Purified sequencing reaction products were electrophoresed using an ABI Prism 3130 Genetic Analyzer (Applied Biosystems Inc., USA). Homology searches of the RNA gene sequences were performed using the Gen Bank (www.ncbi.nlm.nih.gov) DNA database with the BLAST program provided online by the DNA Data Bank of Japan.

#### Triiodide analysis

The generation of triiodide was investigated based on the measurement of the absorbance. The behaviour of triiodide generation was estimated qualitatively based on the variation of absorbance at 351 nm. Absorbance measurements were carried out using a UV-2450 UV-visible spectrophotometer.
